# Temporal Trends in Pathogens and Clinical Outcomes of Adult Deep Neck Infections: An 18-Year Multicenter Cohort Study of 12,063 Cases

**DOI:** 10.3390/microorganisms14081826

**Published:** 2026-08-18

**Authors:** Fang-Ching Liu, Ang Lu, Pei-Rung Yang, Yao-Te Tsai, Yao-Hsu Yang, Chia-Yen Liu, Geng-He Chang

**Affiliations:** 1 Department of Pediatrics, Jen-Ai Hospital, Taichung 412, Taiwan; b8301088@gmail.com; 2 Department of Otolaryngology–Head and Neck Surgery, Chang Gung Memorial Hospital, No.8, W. Sec., Jiapu Rd., Chiayi 613, Taiwan; satan54213@gmail.com (A.L.); yaote1215@gmail.com (Y.-T.T.); 3 Department of Traditional Chinese Medicine, Chang Gung Memorial Hospital, Chiayi 613, Taiwan; u9302702@cmu.edu.tw (P.-R.Y.); r95841012@ntu.edu.tw (Y.-H.Y.); 4 School of Traditional Chinese Medicine, College of Medicine, Chang Gung University, Taoyuan 333, Taiwan; 5 Faculty of Medicine, College of Medicine, Chang Gung University, Taoyuan 333, Taiwan; 6 Health Information and Epidemiology Laboratory of Chang Gung Memorial Hospital, Chiayi 613, Taiwan; qchiayen@gmail.com; 7 Graduate Institute of Clinical Medical Sciences, College of Medicine, Chang Gung University, Taoyuan 333, Taiwan

**Keywords:** adult deep neck infection, bacterial etiology, temporal trends, methicillin-resistant *Staphylococcus aureus*, *Streptococcus anginosus* group, empiric antibiotic therapy

## Abstract

Adult deep neck infection (DNI) is a potentially life-threatening condition associated with significant morbidity and mortality. Contemporary large-scale data characterizing long-term microbial evolution and clinical outcome trends in adult DNI remain limited. This study aimed to investigate temporal changes in bacterial pathogens, treatment strategies, and clinical outcomes of adult DNI across an 18-year period. This is a retrospective multicenter cohort study using the Chang Gung Research Database (CGRD) and a de-identified nationwide database from Taiwan’s largest medical system. Hospitalized adult patients (aged ≥18 years) with DNI from 2006 to 2023 were identified and stratified into two consecutive 9-year epochs (Epoch 1: 2006–2014, *n* = 5512; Epoch 2: 2015–2023, *n* = 6551). Demographics, comorbidities, treatment modalities, disease severity, and microbiological profiles were analyzed. Bacterial isolates were evaluated at genus and species levels, with methicillin-sensitive *Staphylococcus aureus* (MSSA) and methicillin-resistant *S. aureus* (MRSA) assessed separately. Among 12,063 adult DNI patients (Epoch 1: *n* = 5512; Epoch 2: *n* = 6551), antibiotic-only treatment increased (77.3% to 83.2%) and surgical intervention decreased (22.7% to 16.9%). Descending necrotizing mediastinitis decreased markedly (2.7% to 0.7%) and in-hospital mortality declined (7.7% to 6.4%). Poly-microbial infections increased substantially (45.3% to 53.7%). Among facultative anaerobic and aerobic isolates, the *Streptococcus anginosus* group (SAG) emerged as a clinically important pathogen. Among anaerobes, Prevotella displaced *Peptostreptococcus* as the dominant genus, with *Peptostreptococcus micros* (*Parvimonas micra*) and *Prevotella buccae* emerging as prominent species. Over 18 years, adult DNI in Taiwan demonstrated significant improvements in clinical outcomes, with marked reductions in mediastinitis and in-hospital mortality. Concurrently, poly-microbial infections increased, and the SAG, *Peptostreptococcus* micros (*Parvimonas micra*), *Prevotella*, and anaerobic organisms emerged as clinically important pathogens, underscoring the need for empiric antibiotic regimens providing broad aerobic and anaerobic coverage. Although microbiological profiles vary geographically, these findings provide a contemporary evidence base to guide empiric antibiotic selection, inform surgical decision-making, and identify high-risk adult patients with DNI.

## 1. Introduction

Deep neck infection (DNI) is a serious infectious condition involving the potential spaces and fascial planes of the cervical region [1]. Despite advances in diagnostic imaging and the widespread use of empiric antibiotic regimens, adult DNI remains associated with considerable morbidity and occasional mortality, particularly in patients with underlying metabolic or immunological comorbidities. Because its clinical presentation is highly variable, prompt identification of the causative pathogens is essential to guide the selection of appropriate antimicrobial therapy [1].

Most prior studies of DNI were conducted more than a decade ago, typically at single institutions and over relatively short observation periods [2,3]. Consequently, these data may no longer reflect the current microbial ecology. Moreover, many earlier investigations reported bacterial isolates in aggregate without analyzing their dynamics or temporal evolution [2,3]. This gap limits understanding of the subtle but clinically important shifts in bacterial ecology that can influence empiric treatment strategies and disease severity.

Microbiological profiles and antibiotic resistance patterns in DNI may also vary significantly by geographic region, as demonstrated by international studies from Thailand [4], Greece [5], China [6], and Central Europe [7], underscoring the need for region-specific epidemiological data.

To address these gaps, we used the Chang Gung Research Database (CGRD), a nationwide multi-institutional medical repository, to examine microbiological and clinical outcome trends over an 18-year period (2006–2023) in adult patients with DNI. By characterizing species-level dynamics and linking them to evolving clinical outcomes, this study provides the most current and comprehensive analysis to date of the changing bacterial epidemiology and management of adult DNI.

## 2. Materials and Methods

### 2.1. Data Source: Chang Gung Research Database (CGRD)

The CGRD is a large-scale, de-identified clinical database established from the electronic health records of the Chang Gung Memorial Hospital (CGMH) system—the largest integrated healthcare network in Taiwan [8]. The database encompasses seven medical centers and regional hospitals located across northern, central, and southern Taiwan, capturing comprehensive inpatient and outpatient information across all major specialties. Owing to its breadth and representativeness, the CGRD serves as a reliable national platform for investigating disease epidemiology and healthcare outcomes [8]. Institutional Review Board approval was obtained from CGMH for this study (Approval No. 202301868B0C601).

### 2.2. Study Population

Between 1 January 2006 and 31 December 2023, data were extracted from the CGRD for all inpatients diagnosed with DNI. Cases were identified according to the International Classification of Diseases, Ninth and Tenth Revision (ICD-9-CM and ICD-10-CM) diagnostic codes corresponding to cellulitis or abscess of oral soft tissues (Ludwig’s angina), parapharyngeal abscess, retropharyngeal abscess, and cellulitis or abscess of the neck (ICD-9-CM: 528.3, 478.22, 478.24, 682.1, and equivalent ICD-10-CM codes implemented after October 2015) [1]. An additional inclusion criterion required the presence of a head and neck CT scan examination record during the index hospitalization, as documented in the CGRD electronic health records.

The epidemiologic characteristics and clinical management of DNI differ substantially between pediatric and adult populations [9,10]. Children are more often affected by upper respiratory tract-related infections, whereas adults typically present with odontogenic, diabetic, or immunocompromised etiologies and demonstrate greater variability in comorbid conditions and disease severity [11]. Given these distinctions, a separate analysis focusing exclusively on adult DNI was conducted to delineate age-specific microbiological patterns and outcome trends.

Only patients aged 18 years or older who were hospitalized for DNI were included in the present cohort. Those with missing demographic or diagnostic data were excluded. To examine temporal changes in patient characteristics, treatment strategies, and bacterial spectrum, the study population was divided into two time periods: 2006–2014 and 2015–2023. Demographic variables, comorbidities, treatment modalities (including antibiotic therapy, surgical drainage, and tracheostomy), clinical outcomes (such as mediastinitis, length of hospital stay, and mortality), and bacterial culture profiles were compared between these two periods to evaluate long-term trends in disease presentation, management, and prognosis.

### 2.3. Medical Comorbidities

Comorbidities were identified using the ICD-9-CM and ICD-10-CM codes obtained from both inpatient and outpatient records in the Chang Gung Research Database. A condition was classified as a comorbidity if it was documented at least once during hospitalization or on three or more separate outpatient visits before the index admission for DNI.

For the adult cohort, comorbidities of interest included diabetes mellitus (DM), chronic kidney disease (CKD), and autoimmune disorders such as rheumatoid arthritis (RA), systemic lupus erythematosus (SLE), and Sjögren syndrome (SS) (ICD-9-CM codes 250, 585–586, 403–404, 446.0–446.7, 696.0–696.1, 710.0–710.4, 714.0–714.4, and corresponding ICD-10-CM codes after October 2015) [1]. These comorbidities were selected because they represent well-established risk factors for DNI.

### 2.4. Bacterial Spectra

Adult DNIs are generally polymicrobial in origin, reflecting the diverse microbiota of the upper aerodigestive tract and the frequent presence of odontogenic or systemic comorbid sources [1]. The predominant organisms typically include facultative anaerobes such as Streptococcus spp. and Staphylococcus spp., aerobic Gram-negative bacilli such as Klebsiella and Pseudomonas, and obligate anaerobes including *Prevotella*, *Peptostreptococcus*, and *Veillonella* [1]. Appropriate empiric therapy therefore requires antimicrobial coverage for both facultative/aerobic and anaerobic pathogens.

In this study, all culture-positive isolates from adult DNI cases recorded in the CGRD were classified into two major categories: (1) facultative anaerobic/aerobic bacteria and (2) obligate anaerobes. Detailed analyses were then performed at both the genus and species levels to identify long-term changes in the composition of bacterial flora between 2006–2014 and 2015–2023. Special attention was given to antimicrobial-resistant organisms, particularly methicillin-resistant *Staphylococcus aureus* (MRSA), to delineate temporal shifts in resistance patterns and their clinical implications. Anaerobic specimens were collected during surgical drainage or diagnostic aspiration and transported in pre-reduced anaerobic transport media to minimize oxygen exposure. In the clinical microbiology laboratory, specimens were inoculated onto anaerobic blood agar and incubated under strict anaerobic conditions (5% CO_2_, 10% H_2_, 85% N_2_) at 37 °C for 5–7 days. Organism identification was performed by conventional biochemical testing panels in the earlier study period and progressively supplemented by matrix-assisted laser desorption ionization time-of-flight (MALDI-TOF) mass spectrometry as this technology was adopted across CGMH institutions. The progressive implementation of MALDI-TOF enhanced species-level identification of fastidious anaerobes and likely contributed in part to the observed increase in poly-microbial detection rates between the two epochs.

### 2.5. Therapy Classification

Therapeutic interventions were identified from inpatient records during the index admission for DNI. Patients who received intravenous antibiotic therapy, with or without needle aspiration of the abscess but without definitive surgical drainage, were classified under the antibiotic-only treatment group. Those who underwent incision and drainage or other operative procedures targeting the deep neck spaces were categorized as having received surgical intervention. In addition, patients who underwent tracheostomy were recorded separately to assess the frequency and indications of airway management in adult DNI cases.

### 2.6. Disease Severity and Prognosis Evaluation

Disease severity at presentation was assessed using laboratory parameters obtained upon hospital admission, including white blood cell (WBC) count, C-reactive protein (CRP) level, and the proportion of band forms. Clinical indicators of severe disease were further evaluated, such as the need for intensive care unit (ICU) admission, tracheostomy, and the occurrence of mediastinal complications. Treatment outcomes were analyzed based on in-hospital mortality during DNI management. For patients who died, additional assessments determined whether mediastinal involvement or tracheostomy had been associated with the fatal course, providing a more comprehensive understanding of prognostic factors in adult DNI.

### 2.7. Statistical Analysis

Comparative analyses between the two study periods (2006–2014 and 2015–2023) were conducted to identify temporal changes in clinical characteristics and outcomes. Categorical variables, including sex, comorbidities, treatment modalities, ICU admission, tracheostomy, mediastinal complications, and in-hospital mortality, were examined using the Pearson chi-square test or Fisher’s exact test as appropriate. Continuous variables, such as age, WBC count, and CRP level, were analyzed using Student’s *t*-test. All statistical computations were performed using SAS software, version 9.4 (SAS Institute, Cary, NC, USA), and a two-tailed *p* value < 0.05 was considered statistically significant. Multivariate logistic regression was performed to identify independent predictors of 3-month mortality. The model included study epoch (2015–2023 vs. 2006–2014 as reference), sex, age (per 10-year increment), and clinically relevant comorbidities and therapeutic variables: diabetes mellitus, chronic kidney disease, Sjögren syndrome, rheumatoid arthritis, surgical drainage, and mediastinal involvement. Results are expressed as crude and adjusted odds ratios (OR) with 95% confidence intervals (CI) and two-sided *p*-values.

## 3. Results

### 3.1. Study Population and Comorbidity Profiles

**Figure 1:**
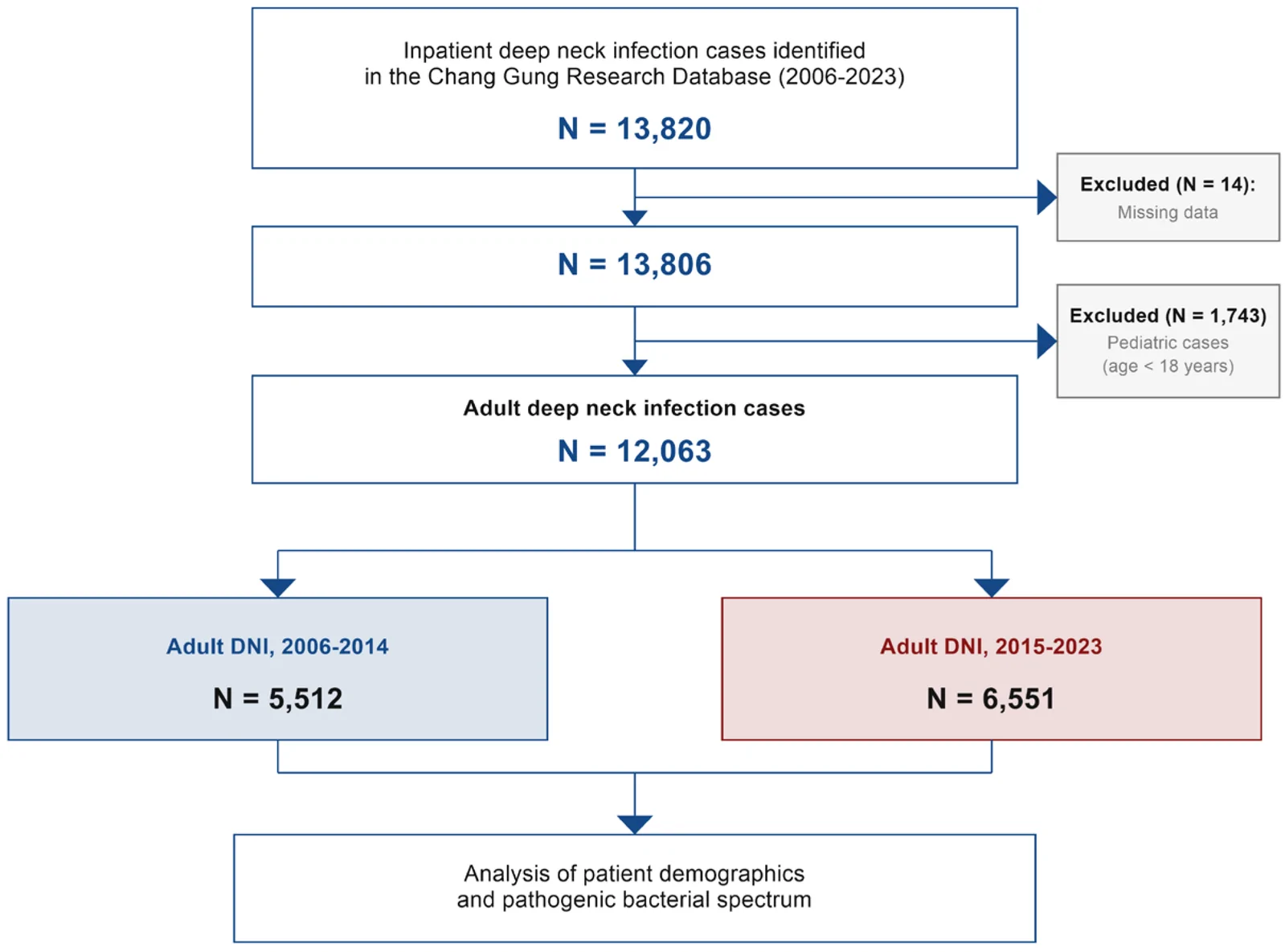
Flowchart of Patient Selection for Adult DNI. Flow diagram illustrating the selection of adult patients with deep neck infection (DNI) identified from the Chang Gung Research Database between 2006 and 2023. Of 13,820 adult and pediatric DNI inpatients, 14 patients with missing data and 1743 pediatric cases were excluded, yielding 12,063 adult patients (Epoch 1 [2006–2014], *n* = 5512; Epoch 2 [2015–2023], *n* = 6551).

Between January 2006 and December 2023, 13,820 adult and pediatric DNI inpatients were identified from the CGRD. After excluding 14 patients with missing data and 1743 pediatric cases, 12,063 adult patients were enrolled and stratified into two consecutive 9-year epochs: Epoch 1 (January 2006–December 2014, *n* = 5512) and Epoch 2 (January 2015–December 2023, *n* = 6551) (Figure 1).

The mean age of the cohort increased significantly from Epoch 1 to Epoch 2 (52.8 ± 16.0 vs. 55.1 ± 16.3 years; *p* < 0.001), while the proportion of male patients declined slightly (67.1% vs. 65.3%; *p* = 0.037) (Table 1). Several comorbidities increased significantly over the study period. The prevalence of chronic kidney disease (CKD) nearly doubled (5.5% vs. 10.0%; *p* < 0.001), and rheumatoid arthritis (RA) (1.1% vs. 1.9%; *p* = 0.001) and Sjögren syndrome (SS) (0.5% vs. 1.2%; *p* < 0.001) also increased. Diabetes mellitus (DM) showed a modest increase (25.7% vs. 27.4%; *p* = 0.044). The prevalences of liver cirrhosis (4.4% vs. 4.9%; *p* = 0.235) and systemic lupus erythematosus (SLE) (0.5% vs. 0.7%; *p* = 0.355) remained stable across epochs.

### 3.2. Treatment Patterns and Clinical Outcomes

The management approach to adult DNI shifted significantly over the study period (Table 2). Antibiotic-only treatment increased from 77.3% to 83.2% (*p* < 0.001), while surgical intervention decreased from 22.7% to 16.9% (*p* < 0.001). The rate of tracheostomy declined from 4.7% to 3.9% (*p* = 0.018), whereas ICU admission rates remained stable (8.5% vs. 8.7%; *p* = 0.682). Descending necrotizing mediastinitis decreased markedly from 2.7% to 0.7% (*p* < 0.001). In-hospital mortality decreased from 7.7% to 6.4% (*p* = 0.005), and mortality associated with mediastinitis declined from 0.4% to 0.1% (*p* < 0.001). The mean length of hospital stay decreased from 12.6 ± 12.7 to 10.9 ± 10.9 days (*p* < 0.001). Admission WBC counts (12.8 ± 6.3 vs. 12.7 ± 6.0 ×10^3^/µL; *p* = 0.267) and CRP levels (101.4 ± 93.8 vs. 100.0 ± 93.0 mg/L; *p* = 0.465) were comparable across epochs, whereas the proportion of band forms declined significantly (4.7 ± 5.4% vs. 3.2 ± 3.7%; *p* < 0.001).

### 3.3. Microbial Composition Patterns

Among the 12,063 adult DNI patients, microbiological cultures were performed in 30.8% during Epoch 1 and 24.9% during Epoch 2. Among culture-tested cases, the positivity rate was similar across epochs (86.5% vs. 87.5%) (Supplementary Figure S1).

**Figure 2:**
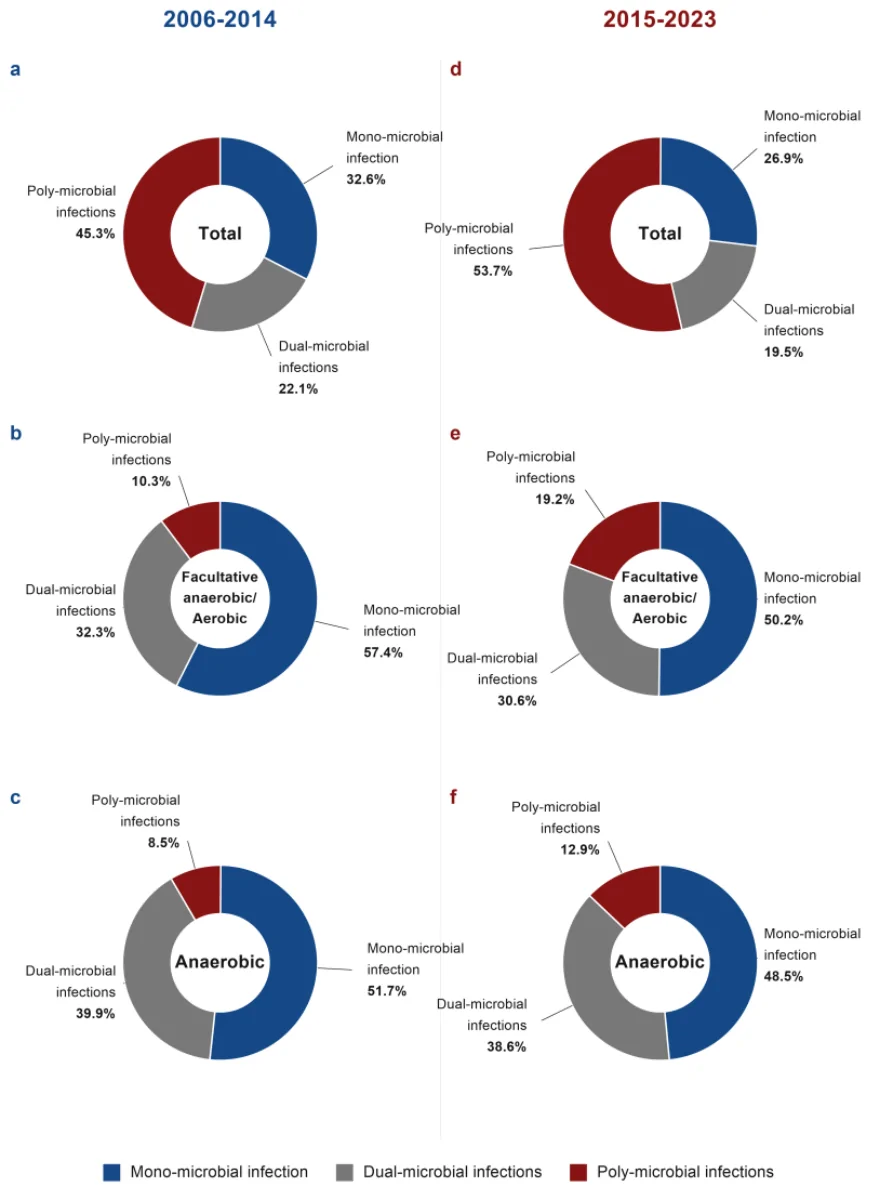
Infection Patterns by Microbial Composition in Adult DNI. Distribution of mono-, dual-, and poly-microbial infections among adult DNI patients during the two study periods (2006–2014 and 2015–2023). (**a**–**c**) represent total, facultative anaerobic/aerobic, and anaerobic infections, respectively, for 2006–2014; (**d**–**f**) represent the same categories for 2015–2023.

Significant shifts in infection complexity were observed between the two epochs (Figure 2). Overall, the proportion of poly-microbial infections increased substantially (45.3% vs. 53.7%), while mono-microbial (32.6% vs. 26.9%) and dual-microbial (22.1% vs. 19.5%) infections decreased correspondingly (Figure 2a,d). Among facultative anaerobic and aerobic isolates, mono-microbial infections declined from 57.4% to 50.2%, whereas poly-microbial infections increased from 10.3% to 19.2%; dual-microbial infections remained relatively stable (32.3% vs. 30.6%) (Figure 2b,e). For anaerobic isolates, the proportion of mono-microbial infections was stable (51.7% vs. 48.5%), while poly-microbial infections increased modestly (8.5% vs. 12.9%) (Figure 2c,f).

### 3.4. Genus-Level Bacterial Ecology

**Figure 3:**
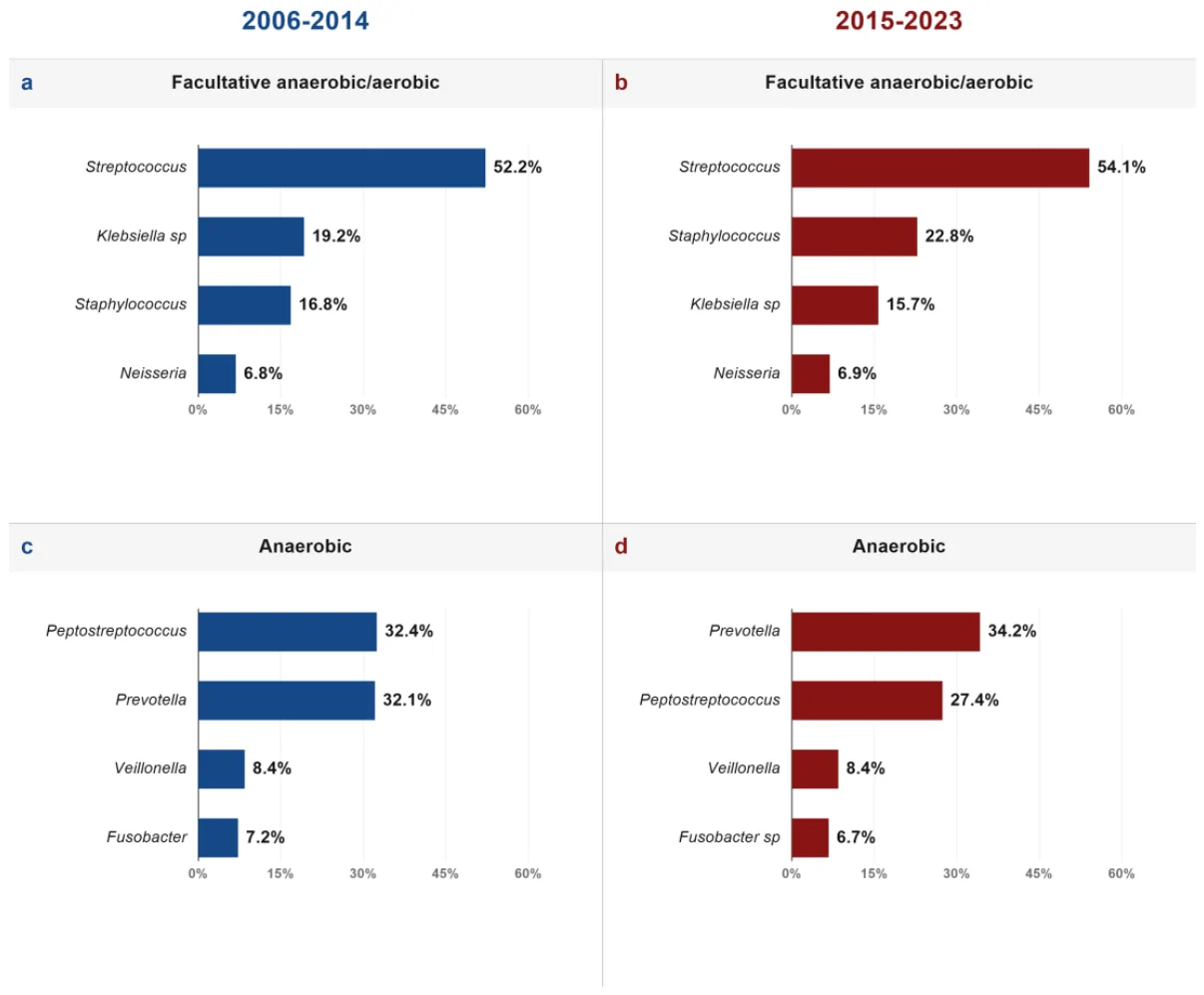
Genus-Level Distribution of Bacterial Isolates in Adult DNI. Bar charts showing the genus-level distribution of facultative anaerobic/aerobic (**a**,**b**) and anaerobic (**c**,**d**) bacterial isolates from adult DNI during the two study periods (2006–2014 and 2015–2023). (**a**,**b**) Among facultative anaerobic/aerobic bacteria, *Streptococcus* remained the dominant genus in both periods (52.2% in 200–2014 and 54.1% in 2015–2023). *Staphylococcus* surpassed *Klebsiella* as the second most prevalent genus in 2015–2023 (22.8% vs. 15.7%), compared with 2006–2014 (16.8% vs. 19.2%). Neisseria remained a minor but persistent genus (6.8% and 6.9%, respectively). (**c**,**d**) Among anaerobes, *Peptostreptococcus* and *Prevotella* co-dominated in 2006–2014 (32.4% and 32.1%, respectively). In 2015–2023, Prevotella emerged as the most prevalent anaerobic genus (34.2%), while *Peptostreptococcus* declined to 27.4%. *Veillonella* remained stable (8.4% in both periods), and Fusobacterium showed a mild decline (7.2% to 6.7%).

Among facultative anaerobic and aerobic bacteria, Streptococcus remained the dominant genus throughout both epochs, with a slight increase in proportion from 52.2% to 54.1% (Figure 3a,b). Notably, *Staphylococcus* surpassed *Klebsiella* as the second most prevalent genus in Epoch 2 (22.8% vs. 15.7%), compared with Epoch 1 (16.8% vs. 19.2%). Neisseria proportions remained stable across epochs (6.8% vs. 6.9%).

Among anaerobic bacteria, Prevotella displaced *Peptostreptococcus* as the most prevalent genus in Epoch 2. *Peptostreptococcus* declined from 32.4% to 27.4%, while Prevotella increased from 32.1% to 34.2% (Figure 3c,d). Veillonella remained unchanged (8.4% vs. 8.4%), and *Fusobacterium* showed a slight decline (7.2% vs. 6.7%).

### 3.5. Species-Level Analysis of Bacterial Cultures

**Figure 4:**
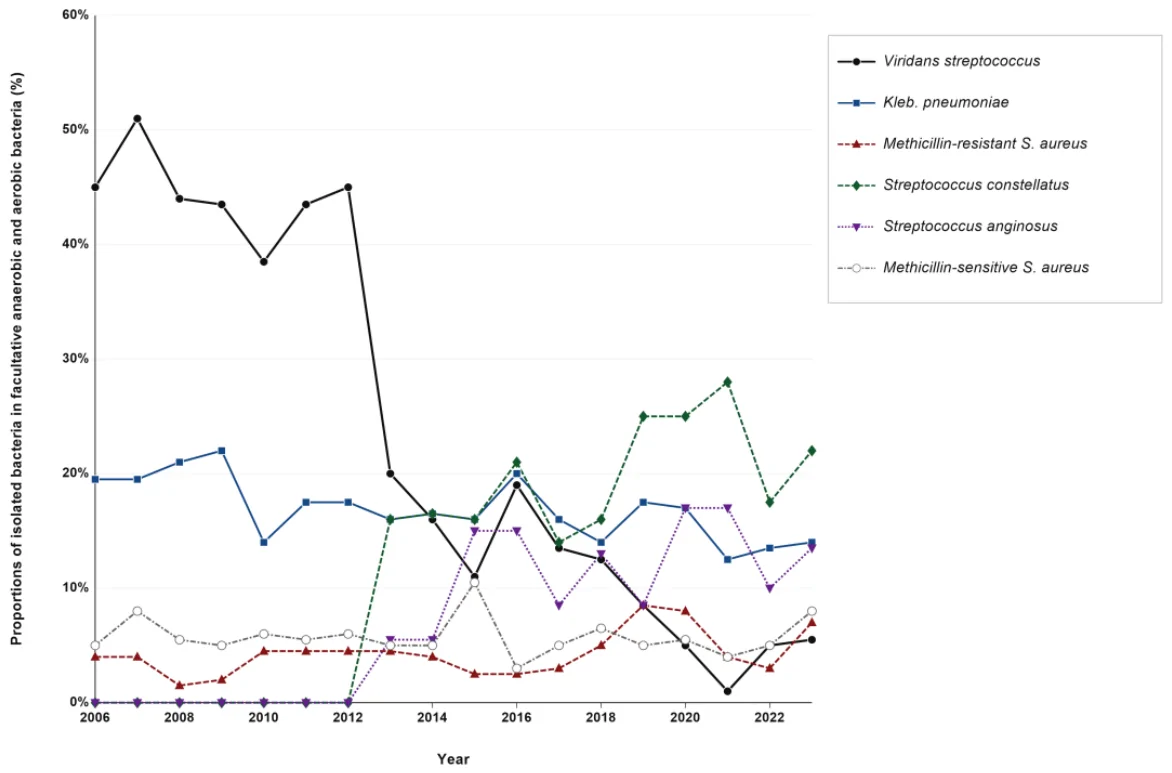
Temporal Trends of Major Facultative Anaerobic and Aerobic Bacterial Species in Adult DNI, 2006–2023. Longitudinal trends showing proportions of the top facultative anaerobic and aerobic bacterial species isolated from adult DNI cases over 18 years. Viridans streptococci predominated in the early period (2006–2014) but declined sharply thereafter. Klebsiella pneumoniae maintained a moderate but steady presence throughout the observation period. Methicillin-resistant *Staphylococcus aureus* (MRSA) and methicillin-sensitive *S. aureus* (MSSA) were consistently detected across all years with only minor fluctuations. Streptococcus constellatus and *Streptococcus anginosus* gradually increased in frequency after 2012.

**Figure 5:**
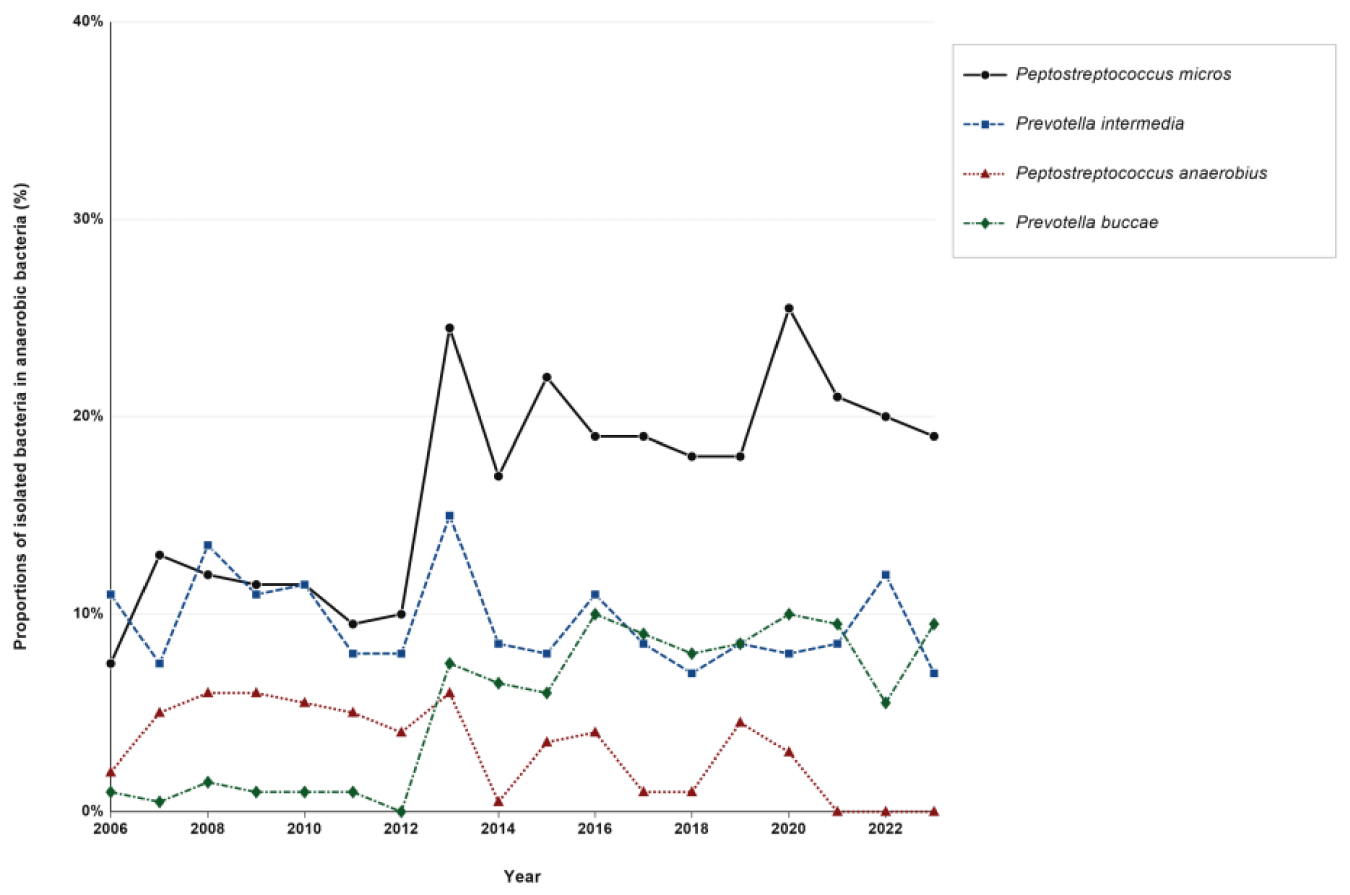
Temporal Trends of Major Anaerobic Bacterial Species in Adult DNI, 2006–2023. Line graph demonstrating the longitudinal distribution of predominant anaerobic bacteria isolated from adult DNI cases across the 18-year study period. *Peptostreptococcus micros* demonstrated a clear rise beginning around 2011 and remained the most frequently identified anaerobic species throughout the later period. Prevotella intermedia showed a relatively stable trend across the entire timeline. Prevotella buccae began to emerge after 2012 and maintained a steady presence through the following decade. In contrast, *Peptostreptococcus anaerobius* gradually declined after 2012, reaching a consistently low frequency in recent years.

Species-level analysis revealed distinct temporal trajectories within both facultative anaerobic/aerobic and anaerobic bacterial groups (Figure 4 and Figure 5).

Among facultative anaerobic and aerobic bacteria, viridans streptococci dominated the early years of the study, comprising nearly half of all isolates before 2014, but declined sharply thereafter. *Klebsiella pneumoniae* maintained a moderate but steady presence throughout the observation period. Both methicillin-resistant *Staphylococcus aureus* (MRSA) and methicillin-sensitive *S. aureus* (MSSA) were consistently detected across all years, with only minor fluctuations and no evident long-term upward or downward trend. In contrast, Streptococcus constellatus and *Streptococcus anginosus* gradually increased in frequency after 2012 (Figure 4).

Among anaerobic bacteria, *Peptostreptococcus micros* demonstrated a clear rise beginning around 2011 and subsequently remained the most frequently identified anaerobic species throughout the later period of the study. Prevotella intermedia showed a relatively stable trend across the entire timeline, maintaining a consistent proportion without significant change. Prevotella buccae began to emerge after 2012 and subsequently maintained a steady presence through the following decade. In contrast, *Peptostreptococcus anaerobius* gradually declined after 2012, reaching a consistently low frequency in recent years (Figure 5).

### 3.6. Factors Associated with In-Hospital Mortality

A total of 12,063 adult DNI patients were included in the mortality analysis, comprising 11,223 survivors and 840 deaths (Table 3). The proportion of patients undergoing surgical intervention was lower in the death group (9.3%) compared with survivors (20.3%; *p* < 0.001). Conversely, tracheostomy and ICU admission were markedly more frequent among non-survivors (8.1% vs. 4.0% and 23.5% vs. 7.5%, respectively; both *p* < 0.001). Descending necrotizing mediastinitis occurred more often in patients who died (3.1%) than in those who survived (1.5%; *p* < 0.001). Regarding microbiological patterns, no significant difference in MRSA detection was identified between the two groups (0.5% vs. 0.7%; *p* = 0.478). However, among facultative anaerobic and aerobic infections, mono-microbial infections were less frequent in non-survivors (5.6%) compared with survivors (11.5%; *p* < 0.001).

In terms of treatment timing, the time to surgery did not differ significantly between non-survivors and survivors (7.9 ± 14.2 vs. 5.9 ± 15.4 days; *p* = 0.256), but time to tracheostomy was shorter in non-survivors (9.1 ± 13.9 vs. 13.6 ± 22.2 days; *p* = 0.024). Laboratory findings further distinguished the two groups: non-survivors had significantly higher ALT (53.6 ± 61.2 vs. 35.5 ± 37.0 U/L; *p* < 0.001), creatinine (1.7 ± 1.8 vs. 1.1 ± 1.0 mg/dL; *p* < 0.001), CRP (146.8 ± 100.4 vs. 97.1 ± 91.8 mg/L; *p* < 0.001), and WBC counts (17.0 ± 8.9 vs. 12.5 ± 5.8 ×10^3^/µL; *p* < 0.001), together with a higher proportion of band forms (6.5 ± 7.3% vs. 3.5 ± 3.9%; *p* < 0.001). Serum albumin was significantly lower in the death group (3.0 ± 0.7 vs. 3.6 ± 0.7 g/dL; *p* < 0.001).

On multivariate logistic regression, the 2015–2023 epoch was independently associated with significantly lower 3-month mortality compared with 2006–2014 (adjusted OR 0.71, 95% CI 0.61–0.82, *p* < 0.001; Table 4). Independent predictors of higher 3-month mortality included male sex (adjusted OR 2.10, 95% CI 1.77–2.50, *p* < 0.001), older age per 10-year increment (adjusted OR 1.37, 95% CI 1.31–1.45, *p* < 0.001), chronic kidney disease (adjusted OR 1.74, 95% CI 1.40–2.16, *p* < 0.001), mediastinal involvement (adjusted OR 2.41, 95% CI 1.54–3.76, *p* < 0.001). Surgical drainage was independently and strongly protective against 3-month mortality (adjusted OR 0.32, 95% CI 0.25–0.41, *p* < 0.001), underscoring the critical role of timely source control. Diabetes mellitus, Sjögren syndrome, and rheumatoid arthritis were not independently associated with 3-month mortality after adjustment. Complete results are presented in Table 4.

## 4. Discussion

This 18-year population-based analysis of 12,063 adult DNI patients from the CGRD provides a comprehensive temporal characterization of microbial ecology and clinical outcomes across two consecutive 9-year epochs. Three principal trends emerged. First, a growing burden of immunocompromising comorbidities—particularly chronic kidney disease (CKD), rheumatoid arthritis (RA), and Sjögren syndrome (SS)—coincided with a substantial shift toward greater infection complexity, with poly-microbial infections increasing from 45.3% to 53.7%. Second, both the facultative anaerobic/aerobic and the anaerobic communities underwent distinct realignments, including the emergence and proliferation of the *Streptococcus anginosus* group (SAG), the displacement of *Peptostreptococcus* by Prevotella as the dominant anaerobic genus, and the emergence of Prevotella buccae. Third, despite these shifts toward greater host vulnerability and microbial complexity, in-hospital mortality improved significantly and descending necrotizing mediastinitis declined markedly [12].

### 4.1. Comorbidity Trends and the Expanding Immunocompromised Host Population

The near-doubling of CKD prevalence (5.5% to 10.0%) and concurrent increases in RA (1.1% to 1.9%) and Sjögren syndrome (0.5% to 1.2%) in our adult DNI cohort likely reflect Taiwan’s aging population and the country’s disproportionately high burden of end-stage renal disease globally. Prior population-based evidence from our group demonstrated that end-stage renal disease is an independent risk factor for DNI and is associated with distinct bacteriological profiles and worse clinical outcomes [13]. The immune dysfunction inherent to CKD—encompassing impaired neutrophil chemotaxis, reduced T-cell function, and complement pathway dysregulation—likely contributes both to increased susceptibility to cervicofascial infection and to impaired resolution of established disease. The parallel rises in RA and SS likely reflect demographic aging combined with the widening use of immunosuppressive and biologic disease-modifying antirheumatic drugs; increased DNI susceptibility in RA patients has been documented in population-based real-world evidence. Notably, DM prevalence remained modestly elevated across both epochs (25.7% vs. 27.4%), consistent with Taiwan’s established epidemic of type 2 diabetes. Collectively, these comorbidity trends indicate that contemporary adult DNI increasingly occurs in immunologically vulnerable hosts, with direct implications for the selection of empiric antibiotic therapy, intensity of monitoring, and the threshold for operative intervention.

### 4.2. Shifting Microbial Complexity

The most prominent microbiological finding in our study was a sustained and substantial increase in poly-microbial infection complexity. Overall, poly-microbial infections rose from 45.3% to 53.7%, driven predominantly by a near-doubling of poly-microbial infections among facultative anaerobic and aerobic isolates (10.3% to 19.2%) and a parallel increase among anaerobic isolates (8.5% to 12.9%), with corresponding declines in mono-microbial patterns. These findings are consistent with secular trends reported from European tertiary-center series, where DNI incidence and microbial complexity have increased over time [12]. Several mechanisms may underlie this evolution. The growing proportion of immunocompromised hosts impairs mucosal barrier integrity and local immune clearance, facilitating the establishment of synergistic aerobic-anaerobic polymicrobial communities from the oral flora. Advances in anaerobic culture methodology and the expanding use of matrix-assisted laser desorption ionization time-of-flight (MALDI-TOF) mass spectrometry for species-level identification have also enhanced detection of previously unrecognized organisms in mixed-culture specimens, contributing at least in part to the observed rise in poly-microbial detection rates.

### 4.3. Shifts in Facultative Anaerobic and Aerobic Bacterial Ecology

Among facultative anaerobic and aerobic bacteria, Streptococcus remained the dominant genus throughout both epochs (52.2% and 54.1%, respectively), consistent with the established role of oral streptococci as primary pathogens in odontogenic and pharyngeal DNI [3]. Staphylococcus surpassed Klebsiella as the second most prevalent genus in Epoch 2 (22.8% vs. 15.7%), reversing the Epoch 1 hierarchy (16.8% vs. 19.2%). The historically prominent role of *K. pneumoniae* in Taiwanese adult DNI—particularly among diabetic patients—reflects the known hypervirulence of K1/K2-capsule-expressing strains endemic to Taiwan, which exhibit exceptional resistance to phagocytosis and are strongly associated with monomicrobial abscess-forming phenotypes [14]. The modest decline in Klebsiella prevalence in Epoch 2 may in part reflect improved glycemic control and intensified outpatient diabetic care over the 18-year study period; nevertheless, its sustained moderate presence reinforces the importance of maintaining empiric Gram-negative coverage in adult DNI patients with hyperglycemia or established DM [1]. At the species level, viridans streptococci dominated the early years of the cohort before declining sharply after 2014, while Streptococcus constellatus and *Streptococcus anginosus*—both members of the SAG—increased steadily after 2012. SAG organisms possess distinct virulence attributes that differentiate them from other viridans streptococci: production of hyaluronidase, deoxyribonuclease, and—in the case of Streptococcus intermedius—the pore-forming cytolysin intermedilysin, which collectively promote connective tissue dissolution and enable spread through natural fascial planes [15]. These properties make SAG infections characteristically abscess-forming and locally aggressive, with a documented propensity for trans-fascial extension into the mediastinum. SAG co-infection has been identified as an independent predictor of progression to descending necrotizing mediastinitis in patients with DNI [16], a finding particularly relevant given the concurrent observation in our cohort that mediastinitis declined markedly over the study period—suggesting that earlier surgical intervention and improved cross-sectional imaging-based detection of fascial extension may be attenuating this complication even as SAG prevalence rises.

### 4.4. Shifts in Anaerobic Bacterial Ecology

Among anaerobic bacteria, Prevotella displaced *Peptostreptococcus* as the leading genus in Epoch 2 (Prevotella: 32.1% to 34.2%; *Peptostreptococcus*: 32.4% to 27.4%). Prevotella species are obligate Gram-negative anaerobes that are integral constituents of the subgingival microbiome and among the most frequently isolated organisms in odontogenic infections, where they contribute to tissue destruction through protease production and lipopolysaccharide-mediated inflammation. Their increasing predominance in adult DNI is consistent with a growing proportion of infections originating from periodontal or odontogenic sources. At the species level, Prevotella buccae emerged as a newly prominent isolate after 2012 and maintained a steady presence through the subsequent decade, while Prevotella intermedia showed a relatively stable longitudinal pattern. *Peptostreptococcus micros*—now reclassified as *Parvimonas micra*—demonstrated a clear and sustained rise beginning around 2011, subsequently becoming one of the most consistently identified anaerobic species in the later study period. *Parvimonas micra* is a Gram-positive anaerobic coccus that inhabits the subgingival sulcus and is increasingly recognized as a causative pathogen in deep cervical abscesses and related head and neck space infections [17]. Enhanced detection of this organism in contemporary clinical practice likely reflects the broader implementation of MALDI-TOF mass spectrometry and improved anaerobic culture methods, which have markedly improved species-level discrimination within the previously undercharacterized anaerobic coccal genera. In contrast, *Peptostreptococcus anaerobius* declined consistently after 2012, suggesting competitive displacement or differential susceptibility within the anaerobic microenvironment of the deep neck space.

Geographic variation in DNI microbiology is an important consideration for the interpretation of our findings. Streptococcus species—particularly the *Streptococcus anginosus* group—represent a consistent predominant pathogen across international cohorts, congruent with our data. However, the relative prominence of specific organisms varies regionally: Klebsiella pneumoniae is proportionally more prevalent in Southeast Asian and Chinese multicenter cohorts [4,6], whereas European series demonstrate a greater contribution from viridans streptococci and anaerobic species [5,7]. These regional differences likely reflect variation in host comorbidity profiles, oral microbiome composition, and local antibiotic stewardship practices. Notwithstanding this heterogeneity, the principle of broad-spectrum empiric antimicrobial coverage targeting both aerobic and anaerobic organisms remains universally applicable across geographic settings.

### 4.5. Clinical Outcomes and Prognostic Factors

In-hospital mortality declined from 7.7% to 6.4% across the two epochs, and descending necrotizing mediastinitis—the most life-threatening complication of adult DNI, historically associated with mortality rates exceeding 20%—decreased markedly from 2.7% to 0.7% [18]. These improvements likely reflect advances in cross-sectional imaging enabling earlier delineation of fascial space involvement, standardization of multidisciplinary surgical protocols, and optimization of perioperative and intensive care management. Surgical drainage remains the definitive treatment for adult DNI with abscess formation or evidence of descending spread, and the decision for operative intervention should be based on clinical and radiologic criteria. Our analysis of mortality predictors revealed that non-survivors demonstrated significantly higher admission levels of CRP (146.8 vs. 97.1 mg/L), WBC (17.0 vs. 12.5 ×10^3^/µL), and band form percentages (6.5% vs. 3.5%), consistent with a more profound systemic inflammatory response at presentation. The clinical utility of inflammatory biomarkers—including C-reactive protein, neutrophil-to-lymphocyte ratio, and composite immune-inflammatory indices—for predicting complications and outcomes in DNI has been demonstrated in prospective studies [19]. Hypoalbuminemia (3.0 vs. 3.6 g/dL) and elevated creatinine (1.7 vs. 1.1 mg/dL) were notably associated with in-hospital death, reflecting the compounding burden of pre-existing nutritional deficiency and renal impairment on immune competence and physiologic reserve; this pattern is consistent with prior observations in end-stage renal disease patients with DNI [13]. Elevated ALT (53.6 vs. 35.5 U/L) in non-survivors further suggests that hepatic dysfunction—whether attributable to sepsis-mediated liver injury or underlying comorbid hepatic disease—contributes to adverse outcomes. Critically, the proportion of patients undergoing surgical intervention was significantly lower in non-survivors (9.3%) than in survivors (20.3%). This pattern should not be interpreted as evidence against operative treatment; rather, it reflects the clinical reality that the most critically ill patients may not be surgical candidates at presentation or may deteriorate before operative drainage can be performed—underscoring the imperative for early and systematic evaluation of surgical candidacy before physiologic deterioration renders operative management unfeasible.

Multivariate logistic regression confirmed that the temporal reduction in 3-month mortality persisted after adjustment for potential confounders (adjusted OR 0.71 for 2015–2023 vs. 2006–2014, *p* < 0.001), supporting genuine improvements in care pathways over the 18-year period—including earlier imaging-guided diagnosis, multidisciplinary management, and institutional antibiotic stewardship. Among host-related factors, male sex, advanced age, and chronic kidney disease were independent mortality predictors, consistent with impaired physiological reserve and immune dysfunction. Surgical drainage was independently protective (adjusted OR 0.32), reaffirming the central role of early source control in improving DNI mortality. Mediastinal involvement independently predicted higher mortality (adjusted OR 2.41), reflecting the severity of complicated DNI with caudal extension.

The absolute increase in DNI cases from Epoch 1 (*n* = 5512) to Epoch 2 (*n* = 6551; +18.8%) likely reflects the convergence of several epidemiological forces: (1) progressive population aging and the attendant expansion of the immunocompromised host population, as evidenced by the near-doubling of CKD prevalence and concurrent rises in autoimmune comorbidities; (2) secular trends toward increasing DNI incidence documented in European multicenter series, attributed to aging demographics and broadening immunosuppressive pharmacotherapy [12]; and (3) expansion of CGMH institutional capacity and catchment population over the 18-year observation period.

### 4.6. Antibiotic Resistance Landscape

A temporal comparison of four major antibiotic-resistant pathogens between the two study epochs demonstrated no statistically significant change in prevalence—MRSA (1.1% vs. 1.2%, *p* = 0.522), MDR-AB (0.2% vs. 0.2%, *p* = 0.224), ESBL-KP (0.02% vs. 0%, *p* = 0.457), and CRPA (1.3% vs. 1.1%, *p* = 0.261; Supplementary Table S1)—indicating a stable antibiotic resistance landscape in DNI over the 18-year observation period. These findings suggest that the epidemiological shift toward greater microbial complexity and increasing prevalence of polymicrobial infections has not been accompanied by a parallel escalation in clinically significant antibiotic resistance among the major resistant pathogens in this patient population.

A recent meta-analysis by Lai et al. (2026) reported that MRSA accounts for 19.2% of *S. aureus* isolates in oral and maxillofacial space infections [20], and Tsai et al. (Laryngoscope 2022) demonstrated that MRSA is the leading pathogen in DNI patients with end-stage renal disease, accounting for 25% of isolates [13]. In contrast, the overall MRSA prevalence in our cohort was low and stable across epochs (1.1% vs. 1.2%, *p* = 0.522). This apparent discrepancy likely reflects differences in study population composition, with our cohort encompassing unselected adult DNI patients across all comorbidity strata. Taken together, these data suggest that empiric MRSA-targeted therapy is not routinely warranted in the general DNI population, though it should be considered in high-risk patients—particularly those with end-stage renal disease or other significant immunocompromising conditions.

International series have documented clindamycin resistance rates of 34–47% across aerobic and anaerobic isolates in DNI [21], with multidrug-resistant strains disproportionately prevalent in severe compared with mild disease (12% vs. 1%) [21]. More than two-thirds of DNI cases have been reported to harbor beta-lactamase-producing organisms [6], supporting the preferential use of beta-lactamase inhibitor combinations—such as amoxicillin–clavulanate or piperacillin–tazobactam—as empiric therapy. The high prevalence of clindamycin resistance across international cohorts challenges its use as empiric monotherapy for DNI. Ongoing local susceptibility surveillance is therefore warranted to optimize empiric antibiotic selection for DNI.

The microbiological landscape of DNI described in the present study is necessarily shaped by the limitations of conventional culture-based pathogen detection. Emerging culture-independent molecular diagnostic approaches—including 16S rRNA polymerase chain reaction (PCR) and metagenomic next-generation sequencing—have substantially expanded pathogen detection in DNI specimens, identifying fastidious and obligate anaerobic organisms (including Fusobacterium necrophorum and Actinomyces species) in culture-negative samples; metagenomic sequencing has been reported to identify anaerobic organisms in up to 83% of tested specimens [5,22]. The progressive adoption of MALDI-TOF mass spectrometry across CGMH institutions during the study period likely contributed to the improved species-level identification of *Parvimonas micra* and Prevotella species observed in the later epoch, and future studies integrating molecular diagnostics with culture-based methods will be essential for a more comprehensive characterization of the evolving DNI microbiome.

### 4.7. Limitations

This study has several limitations. First, as a retrospective database-derived cohort, case ascertainment depended on ICD coding accuracy, and residual misclassification of superficial soft tissue infections as DNI cannot be excluded. However, several features substantially mitigate the risk of misclassification: the diagnostic codes employed (ICD-9-CM: 528.3, 478.22, 478.24, 682.1, and equivalent ICD-10-CM codes) have been used in prior validated CGRD-based DNI studies [1]; all included cases represent inpatient primary diagnoses; the case-identification algorithm required a concurrent head and neck CT scan record during the index hospitalization as an explicit inclusion criterion, providing objective case-level confirmation beyond ICD code assignment; all patients received intravenous antibiotic therapy and/or surgical drainage—interventions inconsistent with superficial soft tissue infections; and the microbiological findings are congruent with the international DNI literature, supporting biological plausibility. The CGRD has been formally validated in peer-reviewed literature [8]. Despite these safeguards, residual misclassification of superficial soft tissue infections as DNI cannot be entirely excluded. Second, microbiological cultures were performed in only 30.8% and 24.9% of patients in Epochs 1 and 2, respectively; as cultured patients had significantly more severe clinical presentations, the bacteriological findings presented here reflect the microbiology of severe and surgically managed DNI and may not be generalizable to mild cases. Third, patient-level variables including dental hygiene practices, prior dental procedures, specific antibiotic regimens, and baseline nutritional status were not uniformly captured. Finally, data are derived exclusively from the Chang Gung Medical Foundation system, and generalizability to other geographic or healthcare settings should be considered with appropriate caution.

## 5. Conclusions

This 18-year multicenter cohort study of 12,063 adult DNI patients demonstrates substantial improvements in clinical outcomes alongside evolving microbial complexity. Despite this escalating microbial complexity, clinical outcomes improved across the two epochs, with descending necrotizing mediastinitis declining markedly and in-hospital mortality decreasing significantly. Concurrently, poly-microbial infections increased, the *Streptococcus anginosus* group emerged as a clinically important pathogen, and Prevotella displaced *Peptostreptococcus* as the dominant anaerobic genus. Elevated systemic inflammatory markers, hypoalbuminemia, and renal impairment on admission were notably associated with in-hospital death. These updated epidemiological and microbiological data provide a contemporary evidence base to guide empiric antibiotic selection, inform surgical decision-making, and identify high-risk adult patients requiring intensified management.

## Figures and Tables

**Table 1:** Demographic characteristics and comorbidities of adult patients with deep neck infection in two consecutive epochs (2006–2014 vs. 2015–2023).

	2006–2014		2015–2023		
Variables	*n*	%	*n*	%	*p*-Value ^a^
Total	5512		6551		
Age (mean ± SD) ^b^	52.8 ± 16.0		55.1 ± 16.3		<0.001
Gender					0.037
Male	3699	67.1	4278	65.3	
Female	1813	32.9	2273	34.7	
Comorbidities					
CKD	305	5.5	653	10.0	<0.001
DM	1419	25.7	1793	27.4	0.044
LC	244	4.4	320	4.9	0.235
RA	61	1.1	124	1.9	0.001
SS	29	0.5	78	1.2	<0.001
SLE	29	0.5	43	0.7	0.355

Data are presented as *n* (%) for categorical variables and mean ± SD for continuous variables. ^a^ Pearson’s chi-squared test; ^b^ Student’s *t*-test. A two-sided *p*-value < 0.05 was considered statistically significant. Abbreviations: CKD, chronic kidney disease; DM, diabetes mellitus; LC, liver cirrhosis; RA, rheumatoid arthritis; SD, standard deviation; SLE, systemic lupus erythematosus; SS, Sjögren syndrome.

**Table 2:** Treatment modalities and clinical outcomes of adult patients with deep neck infection in two consecutive epochs (2006–2014 vs. 2015–2023).

	2006–2014		2015–2023		
Variables	*n*	%	*n*	%	*p*-Value ^a^
Total	5512		6551		
Therapy					<0.001
Antibiotic ± aspiration	4261	77.3	5447	83.2	
Surgery	1251	22.7	1104	16.9	
Tracheostomy	261	4.7	253	3.9	0.018
ICU care	468	8.5	570	8.7	0.682
Mediastinitis	149	2.7	46	0.7	<0.001
Prognosis					
Mortality	423	7.7	417	6.4	0.005
Mediastinitis-related mortality	21	0.4	5	0.1	<0.001
Mortality without tracheostomy	388	7.0	384	5.9	0.009
	mean	SD	mean	SD	*p*-value ^b^
Hospitalization (days)	12.6	12.7	10.9	10.9	<0.001
Laboratory variables					
WBC, ×10^3^/µL	12.8	6.3	12.7	6.0	0.267
CRP, mg/L	101.4	93.8	100.0	93.0	0.465
Band form, %	4.7	5.4	3.2	3.7	<0.001

Data are presented as *n* (%) for categorical variables and mean ± SD for continuous variables. ^a^ Pearson’s chi-squared test; ^b^ Student’s *t*-test. A two-sided *p*-value < 0.05 was considered statistically significant. Abbreviations: CRP, C-reactive protein; ICU, intensive care unit; SD, standard deviation; WBC, white blood cell count.

**Table 3:** Clinical characteristics and laboratory findings of adult patients with deep neck infection stratified by in-hospital mortality.

	Alive		Dead		
Variables	*n*	%	*n*	%	*p*-Value ^a^
Total	11,223		840		
Surgery	2277	20.3	78	9.3	<0.001
Tracheostomy	446	4.0	68	8.1	<0.001
ICU care	841	7.5	197	23.5	<0.001
Mediastinitis	169	1.5	26	3.1	<0.001
MRSA	81	0.7	4	0.5	0.478
Facultative anaerobes or aerobes					<0.001
Mono-infection	1295	11.5	47	5.6	
Dual infection	729	6.5	55	6.6	
Poly-infection	343	3.1	26	3.1	
	mean	SD	mean	SD	*p*-value ^b^
Time to surgery (days)	5.9	15.4	7.9	14.2	0.256
Time to tracheostomy (days)	13.6	22.2	9.1	13.9	0.024
Laboratory variables					
ALT, U/L	35.5	37.0	53.6	61.2	<0.001
Albumin, g/dL	3.6	0.7	3.0	0.7	<0.001
Band form, %	3.5	3.9	6.5	7.3	<0.001
Creatinine, mg/dL	1.1	1.0	1.7	1.8	<0.001
CRP, mg/L	97.1	91.8	146.8	100.4	<0.001
WBC, × 10^3^/µL	12.5	5.8	17.0	8.9	<0.001

Data are presented as *n* (%) for categorical variables and mean ± SD for continuous variables. ^a^ Pearson’s chi-squared test; ^b^ Student’s *t*-test. A two-sided *p*-value < 0.05 was considered statistically significant. Abbreviations: ALT, alanine aminotransferase; CRP, C-reactive protein; ICU, intensive care unit; MRSA, methicillin-resistant *Staphylococcus aureus*; SD, standard deviation; WBC, white blood cell count.

**Table 4:** Risk factors for 3-month mortality in deep neck infection.

	Univariate			Multivariate		
Variables	Crude OR	95% CI	*p*-Value	Adjusted OR	95% CI	*p*-Value ^†^
2015–2023 (ref. 2006–2014)	0.82	0.71–0.94	0.005	0.71	0.61–0.82	<0.001
Sex (ref. female)	1.79	1.52–2.12	<0.001	2.10	1.77–2.50	<0.001
Age (per 10-year increase)	1.34	1.28–1.40	<0.001	1.37	1.31–1.45	<0.001
**Comorbidities**						
DM	1.23	1.06–1.44	0.007	0.94	0.80–1.10	0.435
CKD	2.32	1.90–2.84	<0.001	1.74	1.40–2.16	<0.001
SS	1.38	0.72–2.66	0.331	1.56	0.78–3.09	0.208
RA	1.54	0.95–2.49	0.077	1.39	0.84–2.30	0.205
**Therapy**						
Surgical drainage	0.40	0.32–0.51	<0.001	0.32	0.25–0.41	<0.001
**Complications**						
Mediastinal involvement	2.09	1.37–3.18	0.001	2.41	1.54–3.76	<0.001

^†^ Adjusted for sex, age (per 10-year increment), CKD, DM, SS, RA, surgical drainage, and mediastinal involvement. Abbreviations: CI, confidence interval; CKD, chronic kidney disease; DM, diabetes mellitus; OR, odds ratio; RA, rheumatoid arthritis; SS, Sjögren syndrome.

## Data Availability

The original contributions presented in this study are included in the article/Supplementary Material. Further inquiries can be directed to the corresponding author.

## Supplementary Materials

The following supporting information can be downloaded at: https://www.mdpi.com/article/10.3390/microorganisms14081826/s1, Figure S1: Proportion of Bacterial Cultures and Positive Growth in Adult DNI. Pie charts showing the proportion of culture performance and bacterial growth among adult DNI patients during two study periods (2006–2014 and 2015–2023); Table S1: Demographic characteristics in bacterial growth of 2006–2014 and 2015–2023 in DNI patients over 18 years of age.
