# Supplementary material for: Temporal Trends in Pathogens and Clinical Outcomes of Adult Deep Neck Infections: An 18-Year Multicenter Cohort Study of 12,063 Cases
Source: Microorganisms. 2026 Aug 18;14(8):1826. doi: 10.3390/microorganisms14081826 (PMC13515096; doi:10.3390/microorganisms14081826)
Supplement: Supplementary file 1 [file microorganisms-14-01826-s001.zip › microorganisms-4464285-supplementary.pdf]

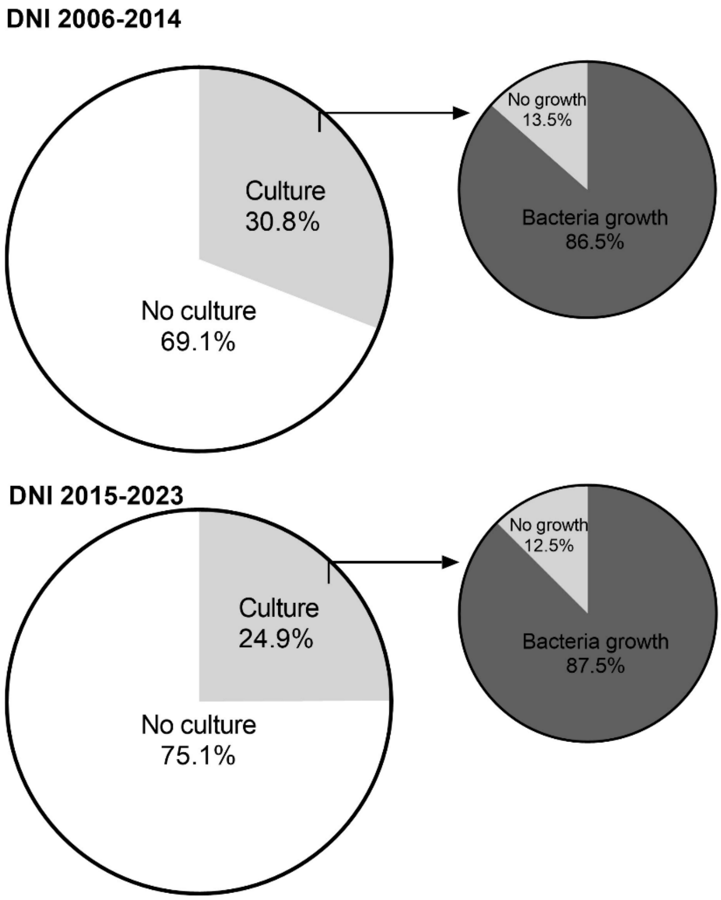

Figure S1: Proportion of Bacterial Cultures and Positive Growth in Adult DNI. Pie charts showing the proportion of culture performance and bacterial growth among adult DNI patients during two study periods (2006–2014 and 2015–2023)

Table S1. Demographic characteristics in bacterial growth of 2006-2014 and 2015-2023 in DNI patients over 18 years of age

		2006-2014		2015-2023	
Num of cases		5512	100.0%	6551	100.0%
Num of cultures		1697	30.8%	1633	24.9%
Num of bacterial growth		1468	26.6%	1429	21.8%
Classification		N	%	N	%
**Facultative anaerobic/GPB**	**Actinomyces sp**	2	0.1	99	6.7
	Actinomyces israelii	1	0.1	0	0.0
	Actinomyces funkei	0	0.0	1	0.1
	Actinomyces graevenitzii	0	0.0	1	0.1
	Actinomyces meyeri	0	0.0	1	0.1
	Actinomyces naeslundii	0	0.0	2	0.1
	Actinomyces neuii	0	0.0	1	0.1

	Actinomyces odontolyticus	0	0.0	45	3.1
	Actinomyces oris	0	0.0	5	0.3
	Actinomyces turicensis	0	0.0	1	0.1
	Actinomyces sp	1	0.1	45	3.1
**Actinobacillus sp**	Actinobacillus sp	1	0.1	0	0.0
	Actinob. actinomycetemcomitans	1	0.1	0	0.0
**Facultative anaerobic/GNB**	**Aeromonas sp**	1	0.1	0	0.0
	Aeromonas sp	1	0.1	0	0.0
**Facultative anaerobic**	**Aggregatibacter sp**	0	0.0	4	0.3
	Aggregatibacter aphrophilus	0	0.0	3	0.2
	Aggregatibacter segnis	0	0.0	1	0.1
**Facultative anaerobic**	**Aerococcus sp**	1	0.1	0	0.0
	Aerococcus	1	0.1	0	0.0
**Facultative anaerobic**	**Brachybacterium sp**	0	0.0	1	0.1
	Brachybacterium sp	0	0.0	1	0.1
**Facultative anaerobic/GPB**	**Bacillus sp**	3	0.2	1	0.1
	Bacillus	3	0.2	1	0.1
**Facultative anaerobic/GNB**	**Compylobacter sp**	0	0.0	7	0.5
	Campylobacter gracilis	0	0.0	1	0.1
	Campylobacter rectus	0	0.0	6	0.4
**Facultative anaerobic/GNB**	**Citrobacter sp**	23	1.6	22	1.5
	Citrobacter diversus	13	0.9	19	1.3
	Citrobacter amalonaticus	2	0.1	0	0.0
	Citrobacter freundii	8	0.5	3	0.2
**Facultative anaerobic/GPB**	**Corynebacterium sp**	12	0.8	29	2.0
	Corynebacterium amycolatum	0	0.0	1	0.1
	Corynebacterium glucuronolyticum	0	0.0	1	0.1
	Corynebacterium jeikeium	2	0.1	0	0.0
	Corynebacterium resistens	0	0.0	1	0.1
	Corynebacterium sp	10	0.7	10	0.7
	Corynebacterium striatum	0	0.0	15	1.0
	Corynebacterium tuberculostearicum	0	0.0	1	0.1
**Facultative anaerobic**	**Capnocytophaga sp**	8	0.5	7	0.5
	Capnocytophaga sp	7	0.5	2	0.1
	Capnocytophaga sputigena	1	0.1	5	0.3
	**Cryptococcus**	1	0.1	0	0.0

	Cryptococcus sp	1	0.1	0	0.0
**Facultative anaerobic/GNB**	**E.coli**	30	2.0	21	1.4
	E.coli	29	2.0	20	1.4
	E.coli-esbl strain	1	0.1	0	0.0
	E.hermannii	0	0.0	1	0.1
**Facultative anaerobic/GNB**	**Eikenella sp**	61	4.2	33	2.2
	Eikenella corrodens	61	4.2	32	2.2
	Eikenella sp	0	0.0	1	0.1
**Facultative anaerobic/GNB**	**Enterobacter sp**	34	2.3	34	2.3
	Enterobacter aerogenes	3	0.2	2	0.1
	Enterobacter cloacae	27	1.8	30	2.0
	Enterobacter agglomerans	1	0.1	0	0.0
	Enterobacter amnigenus	0	0.0	1	0.1
	Enterobacter gergoviae	2	0.1	1	0.1
	Enterobacter sp	1	0.1	0	0.0
**Facultative anaerobic/GPC**	**Enterococcus sp**	26	1.8	42	2.9
	Enterococcus faecalis	19	1.3	30	2.0
	Enterococcus raffinosus	2	0.1	1	0.1
	Enterococcus sp	3	0.2	2	0.1
	Enterococcus avium	0	0.0	4	0.3
	Enterococcus casseliflavus	0	0.0	1	0.1
	Enterococcus durans	1	0.1	0	0.0
	Enterococcus faecium	1	0.1	5	0.3
**Facultative anaerobic**	**Filifactor sp**	0	0.0	1	0.1
	Filifactor alocis	0	0.0	1	0.1
**Facultative anaerobic**	**Gemella sp**	0	0.0	7	0.5
	Gemella haemolysans	0	0.0	1	0.1
	Gemella morbillorum	0	0.0	6	0.4
**Facultative anaerobic**	**Haemophilus sp**	7	0.5	1	0.1
	Haemo.haemolyticus	0	0.0	1	0.1
	Haemo.influenzae	1	0.1	0	0.0
	Haemo.parainfluenzae	5	0.3	0	0.0
	Haemophilus sp	1	0.1	0	0.0
**Facultative anaerobic/GNB**	**Klebsiella sp**	282	19.2	231	15.7
	Kleb.pneumoniae	272	18.5	225	15.3
	Kleb.pneumoniae-esbl strain	1	0.1	0	0.0
	Kleb.oxytoca	9	0.6	3	0.2

	Kleb.ozaenae	1	0.1	0	0.0
	Kleb.pneumoniae-cr strain	0	0.0	1	0.1
	Kleb.variicola	0	0.0	3	0.2
**Facultative anaerobic / GNB**	**Morganella sp**	12	0.8	12	0.8
	Morganella morganii	12	0.8	11	0.7
	Morganella sp	0	0.0	1	0.1
**Facultative_anaerobic**	**Nocardia sp**	1	0.1	0	0.0
	Nocardia sp	1	0.1	0	0.0
**Facultative anaerobic/GNB**	**Proteus sp**	23	1.6	17	1.2
	Proteus mirabilis	20	1.4	16	1.1
	Proteus penneri	1	0.1	0	0.0
	Proteus vulgaris	2	0.1	1	0.1
**Facultative anaerobic/GNB**	**Providencia sp**	2	0.1	1	0.1
	Providencia rettgeri	2	0.1	0	0.0
	Providencia sp	0	0.0	1	0.1
**Facultative anaerobic/GNB**	**Salmonella sp**	9	0.6	8	0.5
	Salmonella enteritidis b	3	0.2	3	0.2
	Salmonella enteritidis d	6	0.4	5	0.3
**Facultative anaerobic/GNB**	**Serratia sp**	14	1.0	10	0.7
	Serratia marcescens	14	1.0	9	0.6
	Serratia marcescens-cr strain	0	0.0	1	0.1
**Facultative anaerobic/GPC**	**Staphylococcus sp**	246	16.8	334	22.8
	Coag(-) staphylococcus	75	5.1	26	1.8
	Staphlococcus aureus	146	9.9	170	11.6
	Methicillin-resistant	58	4.0	77	5.2
	Methicillin-sensitive	88	6.0	93	6.3
	Staph.epidermidis	23	1.6	126	8.6
	Staphlococcus saccharolyticus	5	0.3	0	0.0
	Staphylococcus capitis	1	0.1	9	0.6
	Staphylococcus lugdunensis	3	0.2	5	0.3
	Staphylococcus hominis	0	0.0	5	0.3
	Staphylococcus caprae	0	0.0	2	0.1
	Staphylococcus haemolyticus	3	0.2	12	0.8
	Staphylococcus warneri	0	0.0	1	0.1
**Facultative anaerobic/GPC**	**Streptococcus sp**	767	52.2	794	54.1
	B-streptococcus-like	1	0.1	1	0.1

	Beta-streptococcus group a	11	0.7	8	0.5
	Beta-streptococcus group b	12	0.8	14	1.0
	Beta-streptococcus group c	1	0.1	0	0.0
	Beta-streptococcus group d	15	1.0	6	0.4
	Beta-streptococcus group g	2	0.1	1	0.1
	Beta-streptococcus group non-abd	142	9.7	17	1.2
	Streptococcus anginosus	19	1.3	171	11.6
	Streptococcus constellatus	44	3.0	306	20.8
	Streptococcus dysgalactiae	0	0.0	2	0.1
	Streptococcus intermedius	4	0.3	39	2.7
	Streptococcus mitis	8	0.5	87	5.9
	Streptococcus oralis	2	0.1	58	4.0
	Streptococcus cristatus	0	0.0	2	0.1
	Streptococcus gordonii	1	0.1	10	0.7
	Streptococcus mutans	0	0.0	2	0.1
	Streptococcus parasanguinis	0	0.0	44	3.0
	Streptococcus peroris	0	0.0	2	0.1
	Streptococcus pneumoniae	2	0.1	2	0.1
	Streptococcus salivarius	4	0.3	48	3.3
	Streptococcus sanguinis	0	0.0	11	0.7
	Streptococcus vestibularis	0	0.0	2	0.1
	Viridans streptococcus	596	40.6	121	8.2
**Facultative anaerobic**	**Solobacterium sp**	0	0.0	9	0.6
	Solobacterium moorei	0	0.0	9	0.6
**Facultative anaerobic**	**Vagococcus sp**	0	0.0	1	0.1
	Vagococcus fluvialis	0	0.0	1	0.1
**Aerobic**	**Aerobes sp**	1	0.1	0	0.0
	Aerobes	1	0.1	0	0.0
**Aerobic/GNB**	**Acinetobacter sp**	16	1.1	28	1.9
	Acinetobacter baumannii	8	0.5	16	1.1
	Acinetobacter haemolyticus	1	0.1	0	0.0
	Acineto.baumannii-mdr strain	0	0.0	2	0.1
	Acinetobacter junii	2	0.1	2	0.1
	Acinetobacter nosocomialis	0	0.0	3	0.2
	Acinetobacter nosocomialis-cr strain	0	0.0	1	0.1
	Acinetobacter nosocomialis-mdr strain	0	0.0	1	0.1
	Acinetobacter pittii	0	0.0	2	0.1
	Acinetobacter ursingii	0	0.0	1	0.1
	Acinetobacter sp	5	0.3	3	0.2

**Aerobic**	**Arthrobacter sp**	0	0.0	2	0.1
	Arthrobacter sp	0	0.0	2	0.1
**Aerobic**	**Burkhoderia sp**	2	0.1	8	0.5
	Burkholderia cepacia	1	0.1	6	0.4
	Burkholderia gladioli	0	0.0	1	0.1
	Burkholderia pseudomalleria	1	0.1	1	0.1
**Aerobic**	**Chryseobacterium sp**	0	0.0	1	0.1
	Elizabethkingia meningoseptia	0	0.0	1	0.1
**Aerobic**	Delftia sp	0	0.0	1	0.1
	Delftia acidovorans	0	0.0	1	0.1
**Aerobic**	**Flavobacterium sp**	0	0.0	4	0.3
	Chryseobacterium gleum	0	0.0	1	0.1
	Chryseobacterium indologenes	0	0.0	1	0.1
	Chryseobacterium sp	0	0.0	2	0.1
**Aerobic**	Alcaligenes sp	0	0.0	1	0.1
	Alcaligenes	0	0.0	1	0.1
**Aerobic**	**Listeria sp**	0	0.0	1	0.1
	Listeria monocytogenes	0	0.0	1	0.1
	Listeria sp	0	0.0	1	0.1
**Aerobic**	**Lactobacillus sp**	4	0.3	11	0.7
	Lactobacillus	4	0.3	4	0.3
	Lactobacillus fermentum	0	0.0	2	0.1
	Lactobacillus gasseri	0	0.0	3	0.2
	Lactobacillus paracasei	0	0.0	1	0.1
	Lactobacillus salivarius	0	0.0	2	0.1
**Aerobic**	**Leclercia sp**	0	0.0	1	0.1
	Leclercia adecarboxylata	0	0.0	1	0.1
**Aerobic**	**Micrococcus sp**	1	0.1	1	0.1
	Micrococcus	1	0.1	1	0.1
**Aerobic**	**Moraxella sp**	0	0.0	1	0.1
	Myroides odoratimimus	0	0.0	1	0.1
**Aerobic**	**Neisseria sp**	100	6.8	101	6.9
	Neisseria flavescens	0	0.0	7	0.5
	Neisseria macacae	0	0.0	3	0.2
	Neisseria mucosa	1	0.1	3	0.2
	Neisseria sicca	3	0.2	3	0.2
	Neisseria subflava	5	0.3	12	0.8
	Neisseria sp	91	6.2	75	5.1
**Aerobic/GNB**	**Pseudomonas sp**	75	5.1	72	4.9
	Ps.aeruginosa--cr strain	0	0.0	1	0.1

	Pseudomonas aeruginosa	72	4.9	71	4.8
	Pseudomonas putrefaciens	1	0.1	0	0.0
	Pseudomonas sp	2	0.1	0	0.0
**Aerobic**	**Rothia sp**	0	0.0	24	1.6
	Rothia mucilaginosa	0	0.0	24	1.6
**Aerobic**	**Raoultella sp**	0	0.0	2	0.1
	Rahnella aquatilis	0	0.0	1	0.1
	Raoultella onithinolytica	0	0.0	1	0.1
**Aerobic**	**Stomatococcus sp**	21	1.4	4	0.3
	Stomatococcus sp	21	1.4	4	0.3
**Aerobic/GNB**	**Stenotrophomonas sp**	2	0.1	1	0.1
	Stenotrophomonas maltophilia	2	0.1	1	0.1
**Aerobic**	**Tsukamurella sp**	0	0.0	1	0.1
	Tsukamurella paurometabola	0	0.0	1	0.1
**Anaerobic/GPB**	**Atopobium sp**	0	0.0	27	1.8
	Atopobium parvulum	0	0.0	18	1.2
	Atopobium sp	0	0.0	10	0.7
**Anaerobic**	**Acidaminococcus sp**	0	0.0	1	0.1
	Acidaminococcus sp	0	0.0	1	0.1
**Anaerobic**	**Alloprevotella sp**	0	0.0	1	0.1
	Alloprevotella sp	0	0.0	1	0.1
**Anaerobic**	**Anaerococcus sp**	0	0.0	1	0.1
	Anaerococcus sp	0	0.0	1	0.1
	**Bulledia sp**	0	0.0	1	0.1
	Bulleidia sp	0	0.0	1	0.1
**Anaerobic/GNB**	**Bacteroides sp**	40	2.7	63	4.3
	Bacteroides fragilis	17	1.2	17	1.2
	Bacteroides pyogenes	0	0.0	13	0.9
	Bacteroides thetaiotaomicron	7	0.5	10	0.7
	Bacteroides distasonis	0	0.0	1	0.1
	Bacteroides gracilis	1	0.1	1	0.1
	Bacteroides nordii	0	0.0	1	0.1
	Bacteroides ovatus	0	0.0	5	0.3
	Bacteroides uniformis	1	0.1	1	0.1
	Bacteroides ureolyticus	1	0.1	0	0.0
	Bacteroides vulgatus	1	0.1	2	0.1
	Bacteroides sp	13	0.9	15	1.0
**Anaerobic/GPB**	**Bifidobacterium sp**	0	0.0	12	0.8
	Alloscardovia omnicolens	0	0.0	4	0.3
	Bifidobacterium dentium	0	0.0	7	0.5

	Bifidobacterium sp	0	0.0	1	0.1
**Anaerobic**	**Bilophila sp**	1	0.1	4	0.3
	Bilophila sp	0	0.0	3	0.2
	Bilophila wadsworthia	1	0.1	1	0.1
**Anaerobic/GPB**	**Clostridium sp**	8	0.5	1	0.1
	Clostridium perfringens	1	0.1	0	0.0
	Clostridium sordellii	1	0.1	0	0.0
	Clostridium sp	6	0.4	1	0.1
**Anaerobic**	**Dialister sp**	0	0.0	24	1.6
	Dialister micraerophilus	0	0.0	4	0.3
	Dialister pneumosintes	0	0.0	20	1.4
**Anaerobic**	**Eggerthia sp**	0	0.0	14	1.0
	Eggerthia catenaformis	0	0.0	13	0.9
	Eggerthia sp	0	0.0	1	0.1
**Anaerobic/GNB**	**Fusobacter sp**	106	7.2	98	6.7
	Fusobacter necrophorum	15	1.0	17	1.2
	Fusobacter nucleatum	65	4.4	27	1.8
	Fusobacter gonidiaformans	0	0.0	3	0.2
	Fusobacter periodonticum	0	0.0	2	0.1
	Fusobacter varium	3	0.2	0	0.0
	Fusobacterium sp	25	1.7	49	3.3
**Anaerobic**	**Lachnoanaerobaculum sp**	0	0.0	2	0.1
	Lachnoanaerobaculum orale	0	0.0	2	0.1
**Anaerobic**	**Megasphaera sp**	0	0.0	1	0.1
	Megasphaera micronuciformis	0	0.0	1	0.1
**Anaerobic**	**Olsenella sp**	0	0.0	2	0.1
	Olsenella uli	0	0.0	2	0.1
**Anaerobic/GPC**	**Peptostreptococcus sp**	475	32.4	402	27.4
	Peptostreptococcus anaerobius	67	4.6	27	1.8
	Peptostreptococcus asaccharolyticus	5	0.3	0	0.0
	Peptostreptococcus prevotii	1	0.1	0	0.0
	Peptostreptococcus indolicus	0	0.0	1	0.1
	Peptostreptococcus intermedius	1	0.1	0	0.0
	Peptostreptococcus magnus	12	0.8	19	1.3
	Peptostreptococcus micros	194	13.2	283	19.3
	Peptostreptococcus oryzihabitans	0	0.0	1	0.1
	Peptostreptococcus sp	244	16.6	87	5.9
**Anaerobic/GNC**	**Porphyromonas sp**	7	0.5	10	0.7
	Porphyromonas gingivalis	0	0.0	5	0.3
	Porphyromonas sp	7	0.5	5	0.3

**Anaerobic/GNB**	**Prevotella sp**	471	32.1	502	34.2
	Prevotella baroniae	0	0.0	25	1.7
	Prevotella buccae	32	2.2	124	8.4
	Prevotella bergensis	0	0.0	1	0.1
	Prevotella bivia	6	0.4	13	0.9
	Prevotella buccalis	0	0.0	1	0.1
	Prevotella denticola	2	0.1	36	2.5
	Prevotella disiens	1	0.1	3	0.2
	Prevotella heparinolytica	1	0.1	5	0.3
	Prevotella histicola	0	0.0	3	0.2
	Prevotella intermedia	142	9.7	134	9.1
	Prevotella jejuni	0	0.0	1	0.1
	Prevotella loescheii	1	0.1	1	0.1
	Prevotella melaninogenica	52	3.5	56	3.8
	Prevotella nanceiensis	0	0.0	19	1.3
	Prevotella nigrescens	0	0.0	15	1.0
	Prevotella oralis	4	0.3	1	0.1
	Prevotella oris	5	0.3	9	0.6
	Prevotella pallens	0	0.0	1	0.1
	Prevotella salivae	0	0.0	1	0.1
	Prevotella veroralis	0	0.0	1	0.1
	Prevotella sp	276	18.8	138	9.4
**Anaerobic/GPB**	**Propionibacterium sp**	73	5.0	60	4.1
	Propion.acnes	52	3.5	41	2.8
	Propion.granulosum	1	0.1	3	0.2
	Propion.avidum	3	0.2	3	0.2
	Propionibacterium sp	17	1.2	15	1.0
**Anaerobic**	**Slackia sp**	0	0.0	71	4.8
	Slackia exigua	0	0.0	68	4.6
	Slackia sp	0	0.0	3	0.2
**Anaerobic/GNC**	**Veillonella sp**	123	8.4	124	8.4
	**Veillonella atypica**	0	0.0	9	0.6
	Veillonella dispar	18	1.2	14	1.0
	Veillonella parvula	13	0.9	75	5.1
	Veillonella sp	93	6.3	30	2.0
**Fungus**	**Candida sp**	11	0.7	22	1.5
	Candida albicans	9	0.6	17	1.2
	Candida glabrata	1	0.1	2	0.1
	Candida orthopsilosis	0	0.0	1	0.1
	Candida parapsilosis	1	0.1	0	0.0

	Candida sp	1	0.1	0	0.0
	Candida tropicalis	1	0.1	3	0.2
**Fungus**	**Yeast-like**	82	5.6	102	6.9
**Fungus**	**Peptoniphilus sp**	0	0.0	7	0.5
	Peptoniphilus harei	0	0.0	7	0.5
**Fungus**	**Mold**	2	0.1	0	0.0
**Fungus**	**Cladosporium sp**	0	0.0	1	0.1
	Cladosporium sp	0	0.0	1	0.1
	**Unknown gnb/gpb**	197	13.4	114	7.8
